# Comparison of efficacy and cost-effectiveness between mobile-bearing and fixed-bearing unicompartmental knee arthroplasty for elderly patients with knee osteoarthritis: a retrospective cohort study

**DOI:** 10.3389/fsurg.2026.1780137

**Published:** 2026-06-26

**Authors:** Liangchun Pan, Linlin Zhang, Zongsheng Yin, Yunchao Shao, Dengpan Yao

**Affiliations:** 1 Department of Joint Surgery, The Third Affiliated Hospital of Anhui Medical University (The First People's Hospital of Hefei), Hefei, China; 2 Department of Orthopedics, The First Affiliated Hospital of University of Science and Technology of China, Hefei, China; 3 Department of Orthopedics, The First Affiliated Hospital of Anhui Medical University, Hefei, China; 4 Department of Orthopedics, Zhongshan Hospital Fudan University, Shanghai, China

**Keywords:** cost benefit analysis, fixed-bearing, mobile-bearing, osteoarthritis, unicompartmental knee replacement surgery

## Abstract

**Objective:**

To compare the mid-term efficacy and cost-effectiveness of unicompartmental knee arthroplasty using a Mobile-Bearing and a Fixed-Bearing in elderly patients with knee osteoarthritis.

**Method:**

This study is a multicenter retrospective cohort study. 502 patients who underwent surgery at four joint centers from July 2020 to July 2023 were included and divided into a Mobile-Bearing Group (*n* = 251) and a Fixed-Bearing Group (*n* = 251) based on the type of prosthesis used. Compare the surgical parameters, visual analog pain scores at multiple postoperative time points (6 weeks, 3 months, 6 months, 1 year, 2 years), knee joint scores, joint range of motion, imaging parameters, complications, and cost-effectiveness between two groups.

**Result:**

The surgical time of the Mobile-Bearing Group was longer than that of the Fixed-Bearing Group, and the difference was statistically significant (t = 8.524, *P* < 0.001). During mid-term follow-up (6 months to 2 years), the VAS score of the Mobile-Bearing Group was significantly lower than that of the Fixed-Bearing Group, while the HSS score, joint range of motion, and hip knee ankle angle were significantly higher than those of the Fixed-Bearing Group (*P* < 0.05). The incidence of liner dislocation is higher in the Mobile-Bearing Group, while there is an increasing trend of progressive wear in the contralateral compartment in the Fixed-Bearing Group. Cost effectiveness analysis shows that the Mobile-Bearing Group has higher direct medical costs and obtains more quality adjusted life years.

**Conclusion:**

Compared with Fixed-Bearing unicompartmental, Mobile-Bearing unicompartmental can provide better pain relief, functional recovery, and patient satisfaction in the mid-term, but it is accompanied by higher medical costs and specific complication risks; Fixed-Bearing unicompartmental is a more economical alternative.

## 1 Introduction

Unicompartmental knee arthroplasty is a mature surgical procedure for treating isolated medial compartment osteoarthritis of the knee joint. Its purpose is to restore knee joint function to a near physiological state by preserving the cruciate ligament and contralateral compartment structure. In current clinical practice, two main prosthetic design concepts are used: Mobile-Bearing and Fixed-Bearing. There are fundamental differences between these two designs in terms of load transfer, polyethylene wear mechanism, and long-term stability (1). Although numerous studies have confirmed that unicompartmental knee replacement surgery can significantly improve joint function and quality of life, the impact of platform selection on medium- and long-term outcomes remains a topic of ongoing debate (2). The design of the Mobile-Bearing can theoretically reduce polyethylene wear and improve joint surface matching, but it may pose a risk of pad dislocation (3). On the contrary, the Fixed-Bearing design simplifies the mechanical environment, which may reduce surgical complexity, but may increase stress at the tibial prosthesis bone interface (4). Biomechanics studies have shown that Mobile-Bearing design may be better in distributing joint contact stress, while Fixed-Bearing design may provide more predictable stability throughout the entire gait cycle (5).

Existing research mostly focuses on short-term imaging results or isolated functional scoring, lacking a systematic comparison of the clinical efficacy and health economics dimensions of the two platforms (6). This evidence gap is particularly prominent in the elderly population, as there are significant differences in the progression rate of joint degeneration, the risk spectrum of complications, and the utilization mode of medical resources among the elderly. The existing data is insufficient to optimize clinical decision-making (7). Previous randomized controlled trials often had small sample sizes or short follow-up periods, failing to fully elucidate the impact of platform design on long-term survival and revision risk (8). In addition, although cost-effectiveness analysis is increasingly valued in the field of joint replacement, prospective cohort studies exploring the relationship between quality adjusted life years and medical costs are still lacking in the field of unicompartmental knee replacement (9). Fixed-Bearing prostheses typically have lower initial costs, but the potential for higher long-term revision rates may push up total expenses (10). In contrast, although Mobile-Bearing prostheses have high technical requirements and expensive implant costs, their potential survival rate advantages may offset some of the economic burden (11). So far, there have been no studies comparing the clinical and economic outcomes of two types of prostheses simultaneously in the same cohort (12). Imaging evaluations indicate that platform selection may affect the progression of contralateral ventricular degeneration (13), and the response patterns of different designs on patient reported outcome measures are not yet clear (14).

This study conducted a retrospective cohort analysis of 502 elderly patients with medial compartment osteoarthritis of the knee joint, comparing the mid-term clinical outcomes and cost-effectiveness of unicompartmental replacement with Mobile-Bearing and Fixed-Bearing. We evaluated the differences in pain control, functional recovery, complications, and quality adjusted life years between two surgical procedures using standardized data from multiple center medical records with a maximum follow-up of 2 years. Calculate the incremental cost-effectiveness ratio to evaluate economic value. This study aims to provide dual evidence of clinical and health economics from a retrospective perspective to support the selection of prosthetic platform design.

## 2 Materials and methods

### 2.1 Research population and design

This study is a multicenter, retrospective cohort study. We reviewed the medical records of 502 elderly patients diagnosed with medial compartment osteoarthritis of the knee joint and undergoing unilateral unicompartmental knee replacement surgery at our hospital and four affiliated joint centers from July 2020 to July 2023. The research plan has been approved by the ethics review committee. Due to the retrospective and observational nature of this study, written informed consent has been officially waived in accordance with national regulations on the use of anonymized secondary clinical data.

Grouping is strictly based on the type of surgical technique received by the patient. The Fixed-Bearing Group included 251 patients who underwent unicompartmental replacement using Fixed-Bearing prostheses. The Mobile-Bearing Group included 251 patients who underwent unicompartmental replacement using Mobile-Bearing prostheses, with a 1:1 allocation ratio between the two groups.

**Figure 1:**
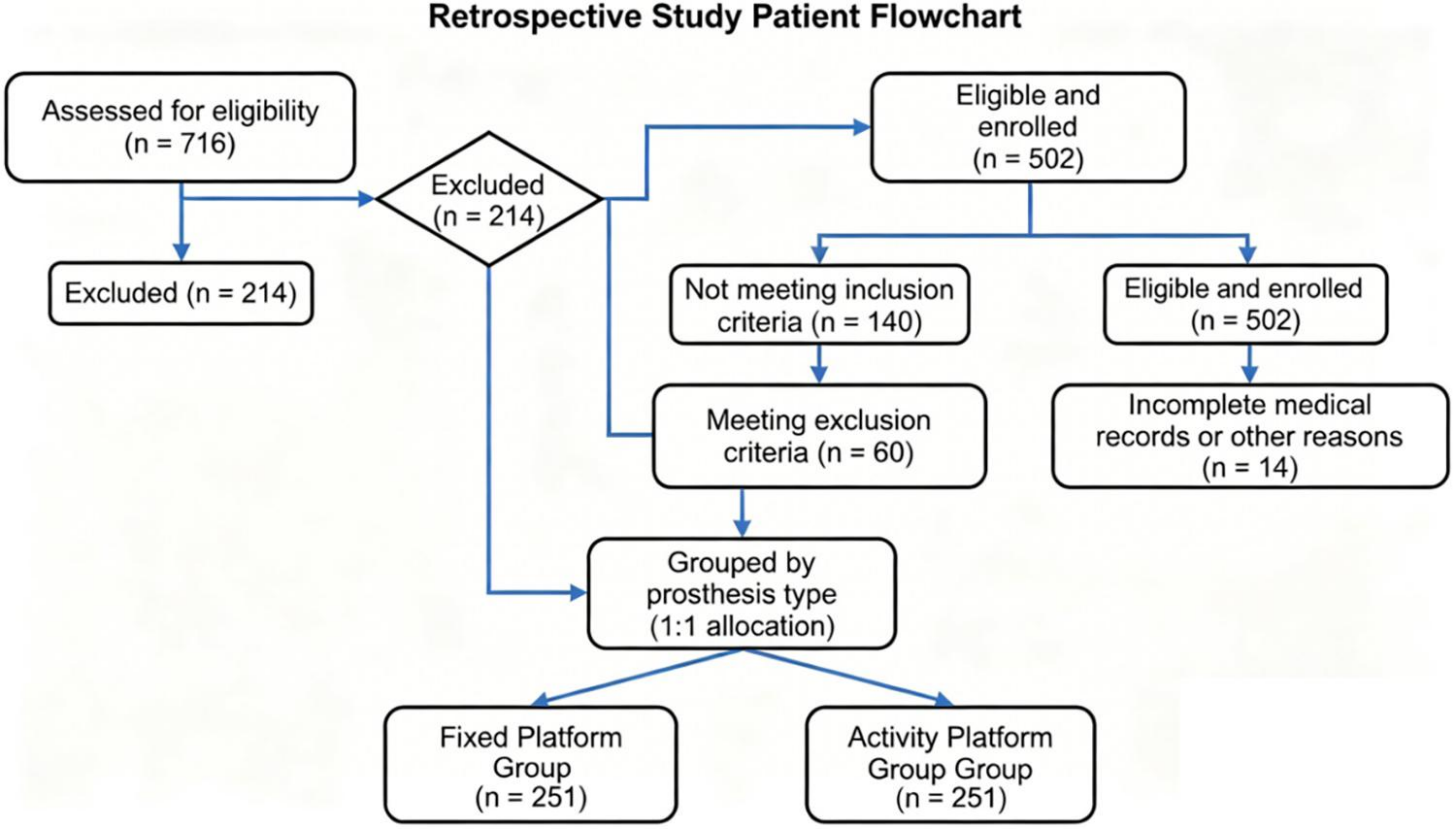
Retrospective study patient flowchart.

Based on post efficacy calculations, the available sample size of 502 cases is considered sufficient for statistical analysis. Based on the existing data of the knee joint score from the US Special Surgery Hospital - preoperative average score of 64.38 (standard deviation 5.94), postoperative average score of 85.19 (standard deviation 4.45) - assuming an effect size f of 0.25. Under the conditions of a statistical power of 80% (*β* = 0.2) and a test level of *α* = 0.05 (two-sided), the minimum sample size required for each group is approximately 248 cases. A total of 502 samples provided sufficient efficacy for testing inter group differences in clinical importance and supported subsequent cost-effectiveness analysis. Retrospective study patient flowchart as shown in Figure 1.

### 2.2 Inclusion and exclusion criteria

Inclusion criteria:

(1) Age range from 60 to 85 years old, gender is not limited.
(2) Diagnosed with primary knee osteoarthritis through clinical and imaging examinations, the lesion is strictly limited to the medial compartment, and the Kellgren Lavrance grading is grade III or IV.
(3) After at least 6 months of standardized conservative treatment (including but not limited to oral nonsteroidal anti-inflammatory drugs, physical therapy, and intra-articular injections), symptoms and pain persist and severely impair daily walking and activity.
(4) Complete cognitive function, able to independently complete self-assessment questionnaires, and committed to cooperating with regular postoperative follow-up evaluations.

Exclusion criteria:

(1) Suffering from secondary arthritis (such as rheumatoid arthritis, psoriatic arthritis, post-traumatic arthritis, or Charcot arthritis).
(2) Anterior or posterior cruciate ligament dysfunction, or significant fixed knee joint deformity (varus/valgus deformity > 15°, or flexion contracture > 15°).
(3) History of open or arthroscopic surgery on the same knee joint.
(4) Individuals with severe heart, lung, liver, and kidney dysfunction, unacceptable anesthesia/surgical risks, or diagnosed with dementia/mental disorders that affect cognitive function and postoperative evaluation compliance.

### 2.3 Implants and devices

All surgeries were performed using domestically produced or imported unicompartmental knee prosthesis systems approved by the China National Medical Products Administration. The Fixed-Bearing Group mainly uses prostheses manufactured by JUST Medical (ZUK system), Waldemar Link (Link Sled), Zhengtian Medical, AK Medical, and Chunli Medical. The Mobile-Bearing Group uniformly uses the Oxford Partial Knee system, including the Biomet Oxford (Zimmer Biomet) and its domestically licensed versions manufactured by AK Medical and Chunli Medical. All components were implanted according to standard surgical procedures.

All surgeries were performed in a vertical laminar flow operating room, using the same model of surgical power system and supporting instruments from Stryker. To improve implantation accuracy, all surgeries were performed with the assistance of a computer navigation system (Boyilai Company) or personalized patient guides based on preoperative 3D CT reconstruction.

Postoperative functional assessment includes objective gait analysis using the G-WALK wireless inertial sensing system (BTS Bioengineering, Italy). All imaging evaluations, including full-length weight-bearing radiographs of both lower limbs and standard lateral radiographs of the knee joint, were performed using a digital x-ray imaging system (Siemens Healthineers, Germany).

### 2.4 Research plan

(1) Research Design: This study is designed as a retrospective, non randomized, observational cohort analysis. The allocation of patients to a Fixed-Bearing Group or a Mobile-Bearing Group is determined by their actual treatment history. The surgical approach recorded for each patient is based on past clinical procedures performed during surgery after standardized shared decision-making between the lead surgeon and the patient. All included surgeries were performed by senior joint surgeons with over 10 years of experience.
(2) Surgical operation and perioperative management: All surgeries are performed by a fixed team of senior doctors to ensure technical consistency. Both groups followed standardized accelerated rehabilitation surgical protocols. The key measures include: not placing a nasogastric tube before surgery; Oral carbohydrate drinks 2 h before surgery; Unified use of laryngeal mask anesthesia combined with adductor block anesthesia; Inject 1.5 grams of cefuroxime intravenously 30 min before skin cutting to prevent infection; Administer 1 gram of tranexamic acid (intravenous infusion combined with local infiltration) during the operation to control bleeding; Postoperative multimodal analgesia regimen (oral administration of celecoxib, and if necessary, intramuscular injection of parecoxib sodium). The two rehabilitation plans are the same, supervised by the same physical therapist team, and weight-bearing walking and joint range of motion exercises begin on the first day after surgery.
  The Fixed-Bearing unicompartmental replacement adopts a minimally invasive medial patellar approach, retaining the knee extension device, followed by precise tibial and femoral osteotomy. The Mobile-Bearing strictly follows the Oxford Phase III surgical technique guidelines for unicompartmental replacement, using minimally invasive approaches that emphasize ligament balance and minimal osteotomy. An independent mobile nurse is responsible for accurately recording the operation time (from skin cutting to wound closure) and calculating blood loss for each surgery, and the nurse is unaware of the subsequent outcome evaluation.
(3) Follow up plan: To ensure objectivity, implement a strict follow-up plan. Regular outpatient follow-up will be arranged at 6 weeks, 3 months, 6 months, 1 year, and 2 years after surgery. During each visit, a dedicated research nurse and a physical therapist are responsible for collecting patient reported outcomes and conducting objective functional measurements, both of whom are unaware of the patient grouping.

### 2.5 Outcome measures

(1) Pain assessment: Visual analog scoring is used for evaluation. The patient is marked on a 10 centimeter straight line, with 0 representing “painless” and 10 representing “the most severe pain imaginable”. Measure the distance from the left end to the marked point (in centimeters, accurate to one decimal place). The higher the rating, the more severe the pain.
(2) Knee joint function score: evaluated using the American Hospital of Special Surgery Knee Joint Score. This percentage scale evaluates pain (30 points), function (22 points), range of motion (18 points), muscle strength (10 points), flexion deformity (10 points), and instability (10 points). The higher the score, the better the knee joint function.
(3) Joint range of motion: The patient lies supine and measures using a standard universal protractor. Align the axis of the protractor with the lateral epicondyle of the femur. The fixed arm is parallel to the femoral shaft, and the moving arm is parallel to the tibial shaft. Record the maximum active flexion and extension angles. The active joint range of motion is defined as the difference between these two angles (in degrees).
(4) Lower limb force line: evaluated on the full-length weight-bearing x-ray of both lower limbs. The hip knee ankle angle is defined by the line connecting the center of the femoral head, the center of the knee joint (usually the tibial crest), and the center of the talus vault. The physiological range is defined as 174° to 180°. Angle <174° indicates inversion, while angle >180° indicates eversion.
(5) Position of prosthesis: Evaluate on standard lateral x-ray of the knee joint. The measurement of posterior tilt angle of tibial prosthesis is the angle between the line connecting the joint surface of the tibial prosthesis and the perpendicular line of the anterior cortical line of the proximal tibia.
(6) Complications recording and grading: Detailed records of all adverse events from surgery to the last follow-up, including superficial/deep infections, symptomatic aseptic loosening, liner dislocation, and progressive wear of the contralateral compartment. The severity is classified using Clavien Dindo grading system. Grade I-II is mild; Grade III and above requiring intervention are defined as major complications.
(7) Cost effectiveness analysis: The direct medical costs of each patient are extracted from the hospital's financial information system, accurately covering prosthesis expenses, anesthesia and surgical expenses, hospitalization drug expenses, hospitalization expenses, and examination fees. Indirect costs are estimated through a questionnaire survey for job losses caused by surgery. The health utility value is derived from the five levels and five dimensions of the European Quality of Life Scale questionnaire, which is converted into utility values using the Chinese utility value system and used to calculate quality adjusted life years. Cost effectiveness analysis starts from the perspective of China's healthcare system. The willingness to pay threshold is set at ¥268,074 per quality adjusted life year, which is three times the per capita GDP of China in 2023, in line with WHO-CHOICE's recommendation for cost-effectiveness thresholds for middle-income countries. Calculate the incremental cost-effectiveness ratio to express the additional cost required for each additional quality adjusted life year obtained by the Mobile-Bearing Group compared to the Fixed-Bearing Group, reported in “yuan/QALY”.
(8) Patient reported outcome: evaluated using knee joint injury and osteoarthritis outcome scores. This tool evaluates five separate dimensions: pain, other symptoms, daily living activity ability, exercise and entertainment function, and knee joint related quality of life. Each dimension rating is converted into a 0–100 point scale, where 0 represents extreme difficulty and 100 represents no problem.

### 2.6 Statistical analysis

Data analysis was conducted using IBM SPSS Statistics 26.0 and R software. Continuous variables that conform to a normal distribution (validated by Shapiro Wilk test) are expressed as mean ± standard deviation and compared using independent sample *t*-test. Non normally distributed continuous variables are represented by median (interquartile range) and compared using Mann–Whitney *U* test. Categorical variables are expressed as examples (percentages) and compared using chi square test or Fisher's exact probability method (when more than 20% of expected frequencies are less than 5).

For the outcome variables of longitudinal measurements (VAS, HSS, ROM), repeated measures analysis of variance was used, with time as the intra subject factor and group as the inter subject factor. Perform Mauchly sphericity test, and if the sphericity hypothesis is violated, use Greenhouse Geisser correction. When significant interaction effects are found, Bonferroni correction is used for simple effects analysis to examine inter group differences at a single time point. Cost effectiveness analysis mainly reports the incremental cost-effectiveness ratio. Conduct sensitivity analysis by constructing a cost-effectiveness acceptable curve through 1,000 resampling using non parametric self-service method. All analyses were considered statistically significant with a bilateral *P* value < 0.05. To correct for potential confounding, a multivariate linear regression model was constructed for continuous outcome measures (2-year VAS, HSS, ROM, KOOS dimensions), with group as the main predictor variable and incorporating the following covariates: age, gender, body mass index, Kellgren Lawrence grading, preoperative values of outcome variables, surgical guidance methods (navigation and personalized guides), and prosthesis brand category (divided into Oxford system, ZUK, Link Sled, and other FB). Multivariate logistic regression was used for binary outcomes such as complications. The model hypothesis is tested through residual diagnosis and variance inflation factor test. Further validate the robustness of the results by conducting subgroup analysis using pre specified guidance methods and comparing the two main FB prostheses with the MB group.

## 3 Results

### 3.1 Comparison of baseline characteristics between two groups of patients

A total of 502 patients were included and allocated, with 251 in the Fixed-Bearing Group and 251 in the Mobile-Bearing Group. The distribution of cases in the four joint centers is as follows: 125 cases (24.9%) in center A, 136 cases (27.1%) in center B, 118 cases (23.5%) in center C, and 123 cases (24.5%) in center D. There were no single center cases exceeding 30% of the total queue, and the proportion of MB and FB patients in each center was comparable (*P* > 0.05). The detailed comparison of preoperative baseline characteristics is shown in Table 1. Among the 15 baseline parameters collected, the two groups were evenly distributed with no statistically significant differences (*P* > 0.05). These comparable parameters include age, gender distribution, body mass index, surgical side, prevalence of complications (hypertension, diabetes), Kellgren Lawrence grading distribution, preoperative visual simulation score pain score, US special surgery hospital knee joint score, knee joint range of motion, hip knee ankle angle, tibial prosthesis posterior inclination, and pain, activities of daily living and quality of life subscale in knee joint injury and osteoarthritis outcome score. The baseline equivalence of demographic characteristics, disease severity, and preoperative functional status between the two groups effectively reduced the potential impact of selection bias on the research results. In the Mobile-Bearing Group, 145 cases (57.8%) used computer navigation, and 106 cases (42.2%) used personalized guides based on preoperative 3D CT reconstruction. In the Fixed-Bearing Group, 148 cases (59.0%) used computer navigation and 103 cases (41.0%) used personalized guides. There was no statistically significant difference in the use of assistive technologies between the two groups (*χ*^2^ = 0.36, *P* = 0.549). To test whether the guidance method changes the treatment effect, interaction terms (group x guidance method) were included in the multivariate model of the main outcome; No significant interaction was found (*P* values for the interaction terms of VAS, HSS, and ROM were all >0.1 after 2 years). In the Mobile-Bearing Group, all 251 patients received the Oxford Partial Knee system or its domestically authorized version: Bonmei Oxford (*n* = 157), Chunli Oxford (*n* = 56), and Aikang Oxford (*n* = 38). The distribution of Fixed-Bearing Groups is as follows: JUST Medical ZUK (*n* = 89), Waldemar Link Sled (*n* = 65), Zhengtian Medical (*n* = 42), JUST Medical (other fixed-bearing designs) (*n* = 30), AK Medical FB (*n* = 18), and Chunli Medical FB (*n* = 7). Given the existence of multiple prosthetic systems within the FB group, we conducted an additional sensitivity analysis limited to the two most commonly used fixed-bearing prostheses (JUST Medical ZUK and Waldemar Link Sled) and the MB group; The main outcomes (VAS, HSS, KOOS) showed no substantial changes, indicating the robustness of the results to the heterogeneity of the prosthesis.

**Table 1:** Comparison of preoperative baseline characteristics between groups.

Characteristic	Fixed-Bearing Group (*n* = 251)	Mobile-Bearing Group (*n* = 251)	Statistic	*P*-value
Age (years, mean ± SD)	68.52 ± 5.73	68.91 ± 6.04	t = 0.742	0.458
Sex [Male, *n* (%)]	112 (44.62)	108 (43.03)	*χ*^2^ = 0.130	0.719
BMI (kg/m^2^, mean ± SD)	26.38 ± 3.41	26.75 ± 3.59	t = 1.184	0.237
Operated Side [Left, *n* (%)]	127 (50.60)	119 (47.41)	*χ*^2^ = 0.510	0.475
Hypertension [*n* (%)]	103 (41.04)	98 (39.04)	*χ*^2^ = 0.207	0.649
Diabetes [*n* (%)]	45 (17.93)	51 (20.32)	*χ*^2^ = 0.464	0.496
K-L Grade [IV, *n* (%)]	138 (54.98)	145 (57.77)	*χ*^2^ = 0.397	0.529
Preop. VAS (points, mean ± SD)	6.85 ± 1.12	6.92 ± 1.08	t = 0.713	0.476
Preop. HSS (points, mean ± SD)	63.91 ± 5.87	64.45 ± 6.02	t = 1.018	0.309
Preop. ROM (°, mean ± SD)	112.36 ± 10.58	113.04 ± 11.27	t = 0.697	0.486
Preop. HKA Angle (°, mean ± SD)	171.28 ± 2.95	171.05 ± 3.11	t = 0.850	0.396
Preop. TCPSA (°, mean ± SD)	7.12 ± 2.36	7.33 ± 2.41	t = 0.986	0.324
Preop. KOOS-Pain (points, mean ± SD)	48.65 ± 8.42	47.92 ± 8.71	t = 0.955	0.34
Preop. KOOS-ADL (points, mean ± SD)	52.18 ± 9.15	51.63 ± 9.44	t = 0.663	0.508
Preop. KOOS-QoL (points, mean ± SD)	35.74 ± 7.28	36.25 ± 7.61	t = 0.767	0.443

### 3.2 Comparison of two surgical parameters and early recovery indicators

**Figure 2:**
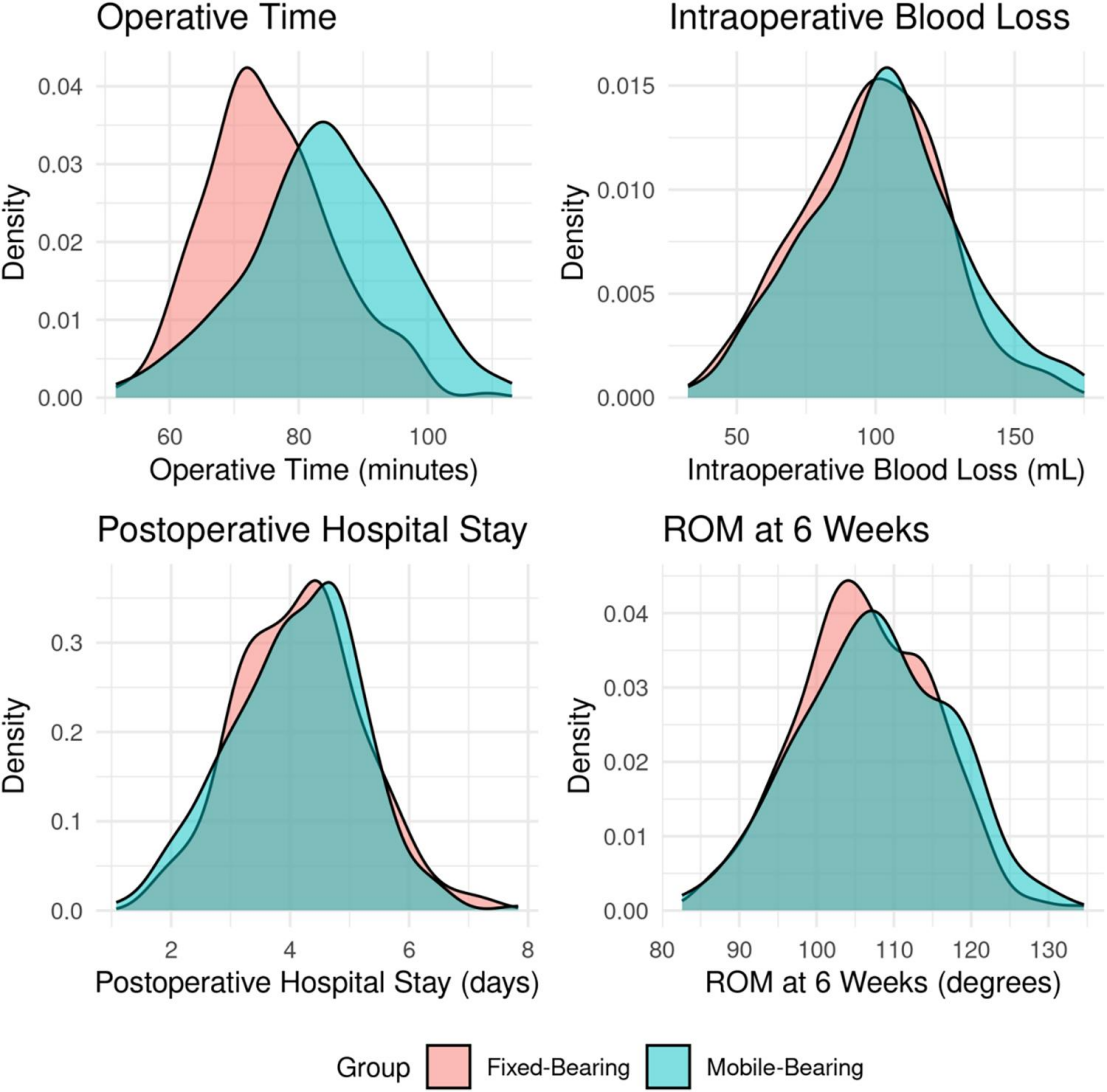
Comparison of surgical data and early recovery metrics between groups.

The surgical parameters and early postoperative recovery data are summarized in Table 2 and Figure 2. The surgical time of the Mobile-Bearing Group was significantly longer than that of the Fixed-Bearing Group (*P* < 0.05). There was no statistically significant difference in intraoperative bleeding between the two groups (*P* > 0.05). The postoperative hospitalization time of the two groups was comparable (*P* > 0.05). At the 6-week follow-up after surgery, both groups showed significant improvement in knee joint range of motion compared to preoperative levels, but there was no significant difference between the two groups at this early time point (*P* > 0.05).

**Table 2:** Comparison of surgical data and early recovery metrics between groups (mean ± SD).

Metric	Fixed-Bearing Group (*n* = 251)	Mobile-Bearing Group (*n* = 251)	Statistic	*P*-value
Operative Time (minutes)	75.64 ± 10.38	82.95 ± 11.72	t = 7.398	<0.001
Intraoperative Blood Loss (mL)	98.75 ± 25.46	102.83 ± 26.91	t = 1.745	0.082
Postop. Hospital Stay (days)	4.12 ± 1.05	4.07 ± 1.11	t = 0.518	0.605
ROM at 6 Weeks (°)	105.82 ± 8.74	106.91 ± 9.25	t = 1.357	0.175

### 3.3 Time variation of postoperative pain and functional score

The dynamic changes in VAS pain scores and HSS knee joint scores at various follow-up time points before and after surgery are detailed in Table 3. Repeated measures ANOVA showed that the main effect of time on VAS and HSS scores was statistically significant (F = 1,253.716, *P* < 0.001; F = 984.325, *P* < 0.001), confirming that both surgical procedures brought significant pain relief and functional improvement. Both outcome measures detected significant interaction between group and time (VAS: F = 4.883, *P* = 0.002; HSS: F = 5.217, *P* = 0.001). Simple effects analysis showed that there was no significant difference in VAS and HSS scores between the two groups at 6 weeks and 3 months after surgery (*P* > 0.05). However, starting from the 6-month follow-up after surgery, the VAS score of the Mobile-Bearing Group remained significantly lower than that of the Fixed-Bearing Group at 6 months, 1 year, and 2 years, while the HSS score was significantly higher than that of the Fixed-Bearing Group (all *P* < 0.05). This mode suggests that the design of the Mobile-Bearing is related to better mid-term pain control and functional recovery.

**Table 3:** Comparison of VAS and HSS scores at preoperative and postoperative time points between groups (mean ± SD).

Outcome	Group	Preoperative	6 Weeks	3 Months	6 Months	1 Year	2 Years
VAS (points)	FB-VAS	6.85 ± 1.12	2.98 ± 0.85	2.15 ± 0.74	1.82 ± 0.69	1.65 ± 0.61	1.59 ± 0.58
	MB-VAS	6.92 ± 1.08	2.91 ± 0.81	2.08 ± 0.70	1.62 ± 0.65^a^	1.41 ± 0.55^a^	1.32 ± 0.52^a^
HSS (points)	FB-HSS	63.91 ± 5.87	78.35 ± 4.26	83.72 ± 3.95	86.48 ± 3.62	87.91 ± 3.41	88.25 ± 3.35
	MB-HSS	64.45 ± 6.02	78.96 ± 4.18	84.51 ± 3.81	88.15 ± 3.48^a^	89.74 ± 3.22^a^	90.13 ± 3.18^a^

a Indicates that there is a statistically significant difference between the groups at this time point (*P* < 0.05).

### 3.4 Postoperative imaging and functional activity results

**Figure 3:**
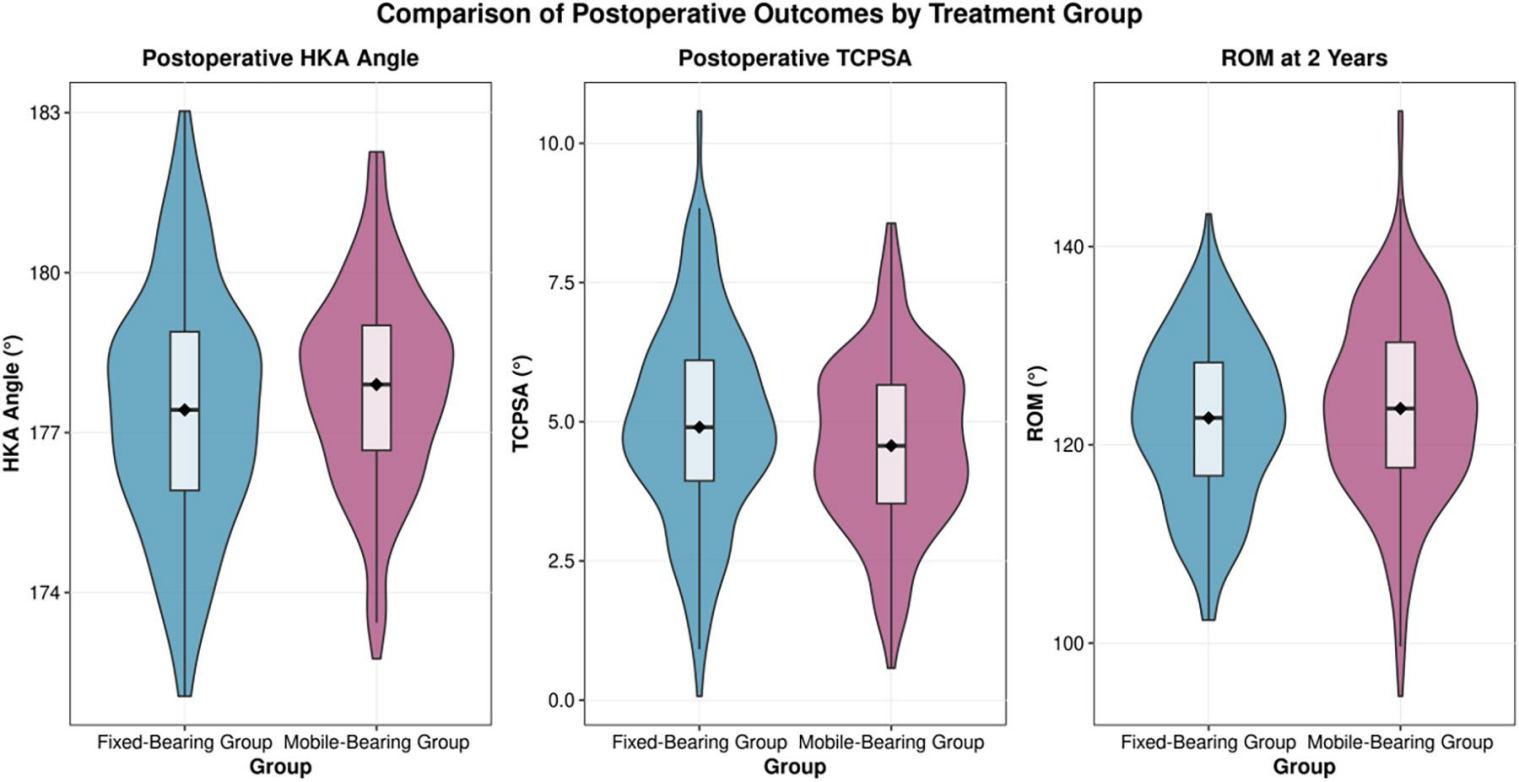
Comparison of radiographic parameters and joint range of motion at 2 years postoperatively.

At 2 years after surgery, the imaging strength line and final knee joint range of motion are shown in Table 4 and Figure 3. The postoperative HKA angle of the Mobile-Bearing Group was closer to the normal physiological range, and the difference was statistically significant compared with the Fixed-Bearing Group (*P* < 0.05). There was no significant difference in the posterior inclination angle between the two groups of tibial prostheses (*P* > 0.05). At the final follow-up, the average knee joint range of motion in the Mobile-Bearing Group was greater, and the difference was statistically significant compared to the Fixed-Bearing Group (*P* < 0.05).

**Table 4:** Comparison of radiographic parameters and joint range of motion at 2 years postoperatively (mean ± SD).

Metric	Fixed-Bearing Group (*n* = 251)	Mobile-Bearing Group (*n* = 251)	Statistic	*P*-value
Postop. HKA Angle (°)	177.15 ± 2.14	177.82 ± 1.96	t = 3.658	<0.001
Postop. TCPSA (°)	4.85 ± 1.72	4.62 ± 1.58	t = 1.560	0.119
ROM at 2 Years (°)	122.58 ± 8.43	124.36 ± 8.91	t = 2.299	0.022

### 3.5 Incidence of complications

The details of complications and Clavien Dindo grading recorded during the 2-year follow-up period are summarized in Table 5. There were 13 cases (5.18%) of complications in the Fixed-Bearing Group and 16 cases (6.37%) in the Mobile-Bearing Group, with no statistically significant difference in the overall incidence of complications (*P* > 0.05). In terms of the types of complications, pad dislocation occurred in 3 patients (1.20%) in the Mobile-Bearing Group, and was not observed in the Fixed-Bearing Group, but the difference did not reach statistical significance (*P* = 0.248). Five cases (1.99%) of progressive wear of the contralateral compartment were observed in the Fixed-Bearing Group, which was higher than one case (0.40%) in the Mobile-Bearing Group, showing a trend of difference (*P* > 0.05). The incidence of periprosthetic infections, symptomatic aseptic loosening, and symptomatic deep vein thrombosis was relatively low and comparable between the groups (*P* > 0.05).

**Table 5:** Comparison of postoperative complications between groups [*n* (%)].

Complication Type	Fixed-Bearing Group (*n* = 251)	Mobile-Bearing Group (*n* = 251)	*χ* ^2^	*P*-value
Periprosthetic Joint Infection	2 (0.80)	1 (0.40)	Fisher*	1.000
Bearing Dislocation	0 (0.00)	3 (1.20)	Fisher*	0.248
Symptomatic Aseptic Loosening	2 (0.80)	1 (0.40)	Fisher*	1.000
Progressive Contralateral Wear	5 (1.99)	1 (0.40)	Fisher*	0.219
Symptomatic Deep Vein Thrombosis	3 (1.20)	4 (1.59)	Fisher*	0.724
Other	1 (0.40)	6 (2.39)	Fisher*	0.123
Total Complications	13 (5.18)	16 (6.37)	0.329	0.566
Clavien-Dindo Grade ≥ III	4 (1.59)	5 (1.99)	Fisher*	1.000

*Fisher's exact test was used.

### 3.6 Cost effectiveness analysis

**Figure 4:**
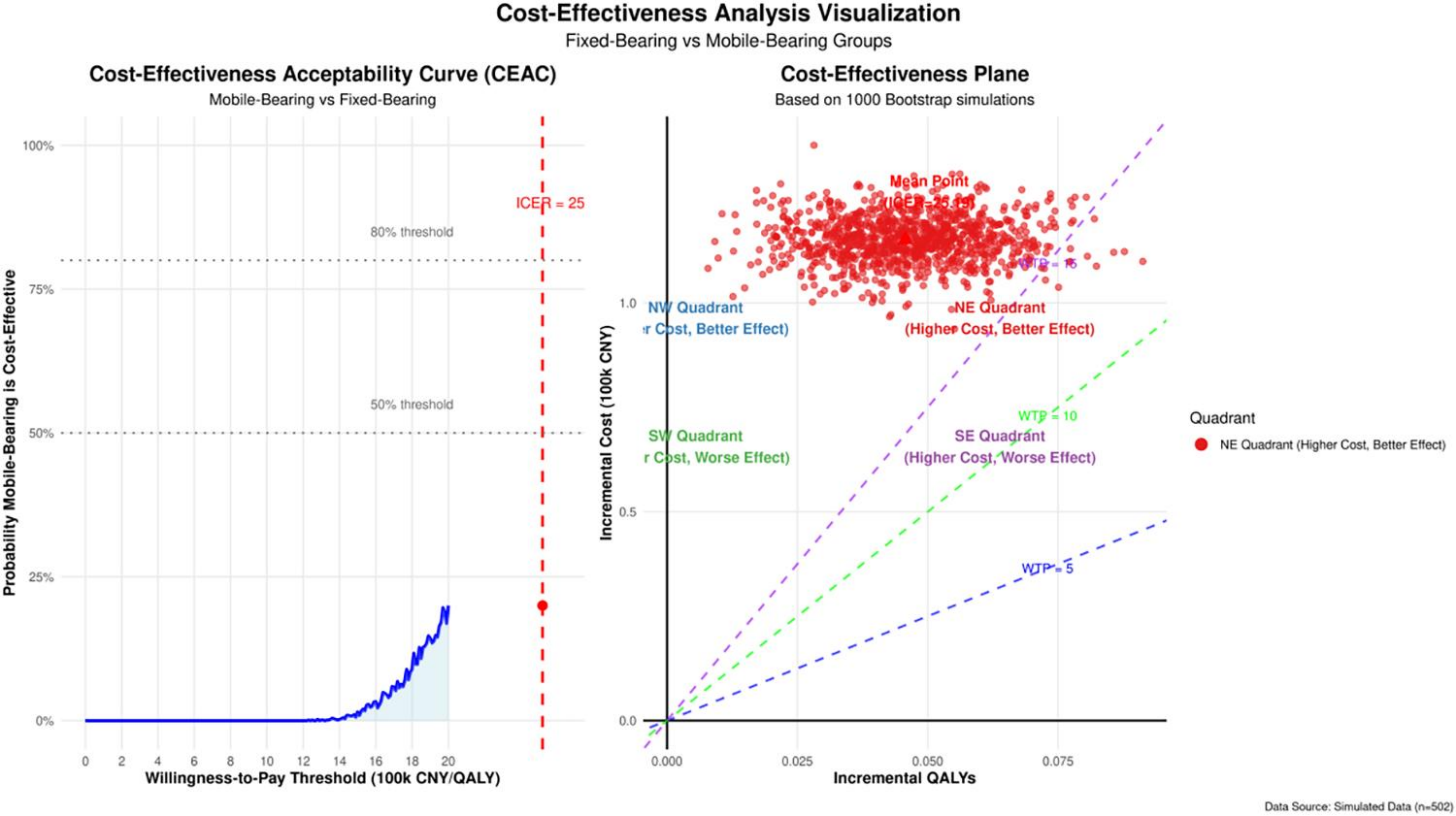
Cost-effectiveness acceptability curve and cost-effectiveness plane.

In the comparison of cost-effectiveness indicators between the Fixed-Bearing Group and the Mobile-Bearing Group over a two-year period, there were significant differences in direct medical costs, total costs, and quality adjusted life years obtained, with the Mobile-Bearing Group having higher values than the Fixed-Bearing Group (*P* < 0.05). However, there was no statistically significant difference in indirect costs between the two groups (*P* > 0.05). Compared with the Fixed-Bearing Group, the incremental cost-effectiveness ratio of the Mobile-Bearing Group is 250,000 yuan per quality adjusted life year. See Table 6 and Figure 4.

**Table 6:** Comparison of cost-effectiveness over 2 years between groups (mean ± SD).

Metric	Fixed-Bearing Group (*n* = 251)	Mobile-Bearing Group (*n* = 251)	Statistic	*P*-value
Direct Medical Cost (10k CNY)	3.92 ± 0.52	4.85 ± 0.61	t = 18.38	<0.001
Indirect Cost (10k CNY)	0.25 ± 0.12	0.23 ± 0.11	t = 1.945	0.052
Total Cost (10k CNY)	4.17 ± 0.58	5.28 ± 0.66	t = 20.02	<0.001
QALYs Gained	1.58 ± 0.15	1.62 ± 0.14	t = 3.089	0.002
ICER (CNY/QALY)	-	250,000	-	-

### 3.7 Patient reported outcome

The detailed assessment of patients' perceived quality of life using KOOS 2 years after surgery is shown in Table 7. The Mobile-Bearing Group had significantly higher scores on all five KOOS subscales than the Fixed-Bearing Group (*P* < 0.05). This indicates that from the patient's perspective, the Mobile-Bearing unicompartmental may provide a better experience in symptom relief, functional improvement, and overall satisfaction with knee related quality of life. In the multivariate linear regression model adjusted for baseline covariates, the Mobile-Bearing Group remained significantly associated with lower 2-year VAS scores (adjusted *β* = −0.22, 95% CI: −0.38 to −0.06, *P* = 0.007) and higher 2-year HSS scores (adjusted *β* = 1.58, 95% CI: 0.42–2.74, *P* = 0.008), consistent with the unadjusted primary analysis results. After sufficient adjustment, the difference between ROM groups was no longer significant (*P* = 0.089). The direction and significance of other outcomes remain unchanged. The subgroup analysis stratified by guidance method and the most commonly used FB prosthesis type did not find significant effect modifications (all interactions *P* > 0.05), confirming the stability of the association.

**Table 7:** Comparison of KOOS scores at 2 years postoperatively between groups (points, mean ± SD).

KOOS Subscale	Fixed-Bearing Group (*n* = 251)	Mobile-Bearing Group (*n* = 251)	Statistic	*P*-value
Pain	85.36 ± 8.15	87.95 ± 7.82	t = 3.633	<0.001
Symptoms	83.71 ± 9.24	86.28 ± 8.76	t = 3.198	0.002
ADL	84.92 ± 8.53	87.11 ± 8.14	t = 2.943	0.003
Sport/Rec	72.58 ± 10.36	76.25 ± 9.87	t = 4.064	<0.001
QoL	75.43 ± 9.72	80.16 ± 9.25	t = 5.585	<0.001

## 4 Discussion

This multicenter retrospective cohort study systematically compared the clinical outcomes and economic efficiency of unicompartmental knee arthroplasty with Mobile and Fixed-Bearings in elderly patients with medial compartment osteoarthritis. The core issue is to clarify the differences in pain relief, functional recovery, quality of life improvement, and cost-effectiveness between the two prosthesis designs, in order to provide information for clinical decision-making. The main results showed that the Mobile-Bearing Group exhibited better pain control, functional scores, and patient reported outcomes during mid-term follow-up. In addition, the group achieved joint mobility and lower limb force line recovery that are closer to physiological states, confirming the inherent biomechanical advantages of its design (15). However, these benefits come with longer surgical times, higher direct medical costs, and a unique spectrum of complications, including the risk of liner dislocation. On the contrary, the Fixed-Bearing Group exhibits relative advantages in terms of economic efficiency and stability of progressive wear in the contralateral compartment (16). Cost effectiveness analysis further reveals that the health benefits brought by Mobile-Bearing prostheses are accompanied by significantly increased costs, and their incremental cost-effectiveness ratio exceeds the commonly referenced willingness to pay threshold. This highlights the extreme importance of individualized treatment strategies, especially in environments with limited medical resources. Based on these results, the necessity of striking a balance between pursuing optimal functional outcomes and balancing related economic burdens is emphasized, providing an evidence-based basis for the practice of joint reconstruction surgery.

Regarding perioperative indicators, the longer operation time of the Mobile-Bearing Group can be attributed to its higher technical requirements for precise soft tissue balance and ensuring pad stability. This surgical plan often requires repeated testing and adjustment during the operation to optimize the trajectory and matching of the polyethylene liner during full range flexion and extension activities, which itself prolongs the operation time (17). There was no significant difference in the calculation of blood loss, indicating that both techniques used minimally invasive approaches combined with standardized hemostatic measures, suggesting that both designs essentially did not cause additional tissue damage, which is consistent with the principle of minimally invasive joint replacement (18). The similar early recovery of activity between the two groups indicates that short-term functional progression is mainly influenced by the quality of postoperative analgesia and the strictness of rehabilitation program implementation, rather than the type of prosthesis itself. However, the design of the Mobile-Bearing may lay the foundation for long-term functional gains. Previous studies have also reported that the duration of unicompartmental surgery on Mobile-Bearings is prolonged but bleeding is controllable, supporting the reliability of this result. However, the clinical significance of this time difference still needs to be considered in conjunction with the technical experience of doctors (19). The underlying mechanism involves the dynamic regulation of ligament tension by the Mobile-Bearing, requiring higher surgical proficiency to avoid under correction and over correction, thereby optimizing postoperative stability (20).

The temporal evolution of mid-term pain and functional scores reveals that the advantages of Mobile-Bearing design continue to emerge six months after surgery. This sustained benefit may stem from its dual condyle bearing design, which more effectively simulates the biaxial kinematics of the natural knee joint and may reduce the peak contact stress of the tibiofemoral joint, thereby reducing the risk of polyethylene wear and soft tissue irritation (21). The steady improvement in functional scores may be related to the rotational freedom allowed by the Mobile-Bearing, which enhances the joint's self adjustment ability during dynamic activities, thereby improving the patient's experience during daily walking and stair climbing (22). Compared with previous literature, most reports support the superiority of Mobile-Bearings in patient reported outcomes, but some argue that these advantages may not be as apparent in elderly populations with lower functional needs - consistent with the relatively high activity level characteristics of patients in this cohort (23). Mechanistically, adaptive movement of the Mobile-Bearing can reduce edge loading and micro motion at the bone implant interface, which may delay the occurrence of painful synovitis and explain why mid-term pain control is better (24). In addition, better mechanical axis recovery may optimize the transmission of force axis, reduce abnormal stress on ligaments around joints, and further consolidate functional benefits.

Imaging evaluation confirmed that the postoperative limb line of the Mobile-Bearing Group was closer to the physiological range of eversion. This advantage stems from the fact that the Mobile-Bearing can continuously adjust the contact points during the buckling process, promoting a more uniform distribution of loads and avoiding the occasional edge stress concentration in the design of Fixed-Bearings (25). The greater range of motion of the terminal knee joint is related to the mismatch of the Mobile-Bearing but the movable matching method. This design reduces the bone impact during terminal flexion, providing more space for movements such as squats (26). Previous studies have observed the advantages of unicompartmental mobility in kinematic recovery, but emphasized that its success is highly dependent on precise prosthesis position and ligament balance; otherwise, the results may be affected (27). The potential mechanisms include: due to the rotatable pad, the frictional resistance between polyethylene and femoral condyle decreases, reducing joint stiffness; meanwhile, the improvement of force lines can indirectly enhance the efficiency of knee extension devices by optimizing the patellar trajectory (28). The synergistic improvement of imaging and functional outcomes highlights the crucial role of prosthesis design in reproducing complex physiological knee joint movements, but long-term follow-up is still needed to confirm the persistence of these findings.

The analysis of complications revealed distinct risk characteristics between two types of prostheses. Pad dislocation is a unique and well documented complication of the Mobile-Bearing Group, often associated with insufficient ligament tension, improper pad size selection, or postoperative changes in joint line height, reflecting the sensitivity of this design to precise soft tissue balance (29). The higher incidence of progressive wear in the contralateral compartment of the Fixed-Bearing Group may be due to stress concentration caused by its relatively fixed pad geometry, which may accelerate cartilage degeneration, especially during dynamic knee joint movement (30). The overall incidence of complications between the two groups is comparable, indicating that both techniques can maintain acceptable safety in the hands of experienced surgeons. However, the differences in the nature of complications require a comprehensive evaluation of the patient's ligament status and expected activity before surgery to guide instrument selection (31). These findings are consistent with international joint registration data, which consistently reports that Mobile-Bearings focus on dislocation rates, while Fixed-Bearings primarily focus on wear related revisions. The relatively low dislocation rate observed by the Mobile-Bearing Group may reflect the technical proficiency of the surgical teams involved in this study (32). Mechanistically, the Mobile-Bearing is prone to subluxation or dislocation when lacking sufficient constraints, while the high matching degree of the Fixed-Bearing, although stable, may transfer stress to the non displaced compartment, thus explaining the observed risk differentiation (33).

Cost effectiveness analysis shows that the higher direct medical costs of the Mobile-Bearing Group are mainly driven by the higher cost of the prosthesis itself and possibly longer operating room time. Although the increase in quality adjusted life years obtained is statistically significant, the magnitude is not significant, resulting in an incremental cost-effectiveness ratio of 250,000 yuan per quality adjusted life year, which is below and close to the commonly cited willingness to pay threshold of 268,074 yuan per quality adjusted life year. This result suggests that from the perspective of the healthcare system, Mobile-Bearing prostheses are near the critical point of conventional cost-effectiveness, and may be economically reasonable for subgroups of people with higher functional requirements or higher evaluations of pain relief. In addition, it should be recognized that cost-effectiveness analysis is limited to a 2-year time frame; Extrapolating over a longer period of time, significant changes in ICER estimates may occur when a higher proportion of liner related complications or revisions may emerge. Therefore, it is necessary to conduct longer-term economic evaluations to determine the persistence of the observed incremental value (34). For ordinary elderly patients, Fixed-Bearing technology provides a more cost-effective option (35). Compared with other health economics studies, this result is consistent with evaluations from other healthcare systems, which also recognize that prosthesis cost is a key factor determining the cost-effectiveness of unicompartmental knee replacement, but also acknowledge that local willingness to pay thresholds must be tailored to specific circumstances (36). One possible underlying mechanism involves the principle of diminishing marginal returns in health utility; the functional advantages of Mobile-Bearing design must be translated into substantial gains in quality of life to justify its additional costs, which may be difficult to achieve for patients with lower activity levels and expectations (37). Sensitivity analysis further suggests that unless the cost of prostheses significantly decreases or the social willingness to pay threshold increases, the widespread adoption of Mobile-Bearing unicompartments still faces economic challenges.

The comprehensive effectiveness of patient reported outcomes reinforces the value of Mobile-Bearing design in patients' subjective experience. Its leading performance on all KOOS subscales (pain, symptoms, activities of daily living, exercise/entertainment, and quality of life) indicates that this design more effectively meets patients' expectations for natural joint sensation and free movement (38). This advantage is likely due to the better adaptability of the Mobile-Bearing design to the complex kinematics of the knee joint, reducing discomfort during specific activities and thereby improving overall life participation (39). Compared with previous studies, most PROMs based studies support the superiority of single condyle Mobile-Bearings, and the differences are more pronounced in patients with higher activity levels, which is consistent with the inclusion criteria of this cohort (40). Mechanistically, an improved biomechanical environment may reduce joint derived muscle suppression, giving patients more confidence in participating in physical activities, while greater joint mobility directly expands the range of activities they can engage in. These factors collectively contribute to a higher quality of life score (41). These findings emphasize the importance of incorporating patient perceived value into outcome evaluation, as it captures health benefits beyond traditional clinical indicators.

Although statistically significant, the absolute inter group differences in some outcome measures are not significant. In terms of 2-year VAS pain scores, the average difference of 0.27 points (MB 1.32 vs. FB 1.59) is lower than the commonly used minimum clinically significant difference of 1.0−1.5 points for chronic musculoskeletal pain, indicating that the observed pain advantage may have critical clinical significance at the individual level. Similarly, the 2-year difference in HSS scores (1.88 points) is close to but not consistently greater than the minimum clinically significant difference of approximately 8–10 points in published HSS knee joint scores, while the difference in KOOS subscales (2–5 points) is also within the range of 8–10 points reported for MCID. Overall, these findings suggest that although the Mobile-Bearing design demonstrates a sustained pattern of statistically superior patient reported outcomes, the clinical relevance of these differences should be interpreted with caution, and treatment decisions should balance cumulative benefits with higher costs and specific complication spectra.

Several limitations of this study deserve careful consideration. Firstly, as a retrospective, non randomized cohort study, it is inherently susceptible to selection bias. The choice of platform design is determined by the chief surgeon after consultation with the patient, and unmeasured confounding factors such as preoperative activity levels, socioeconomic status, or subtle differences in doctors' preferences for specific prosthesis concepts may affect treatment allocation. Although we adjusted for baseline features using multivariate regression and sensitivity analysis, residual confounding cannot be completely ruled out. Retrospective design hinders causal inference, and reported associations should be interpreted with appropriate caution. Secondly, although the 2-year follow-up period is valuable for evaluating mid-term outcomes, it is still insufficient for assessing the long-term survival rate of prostheses and late complications such as aseptic loosening or bone resorption caused by wear particles, which are the more accurate endpoints. In addition, cost analysis is mainly limited to the direct medical perspective of the hospital system. Not including broader social costs such as productivity loss or informal care may underestimate the true economic burden. Future research should extend follow-up to 5 years or more, adopt randomized controlled trial designs to minimize selection bias, integrate a more comprehensive cost-effectiveness analysis framework that covers social costs, and explore predictive models based on patient phenotypes to achieve truly personalized treatment recommendations. Fourthly, the Fixed-Bearing Group includes various prosthetic systems (Jiemai Bangmei ZUK, Link, Zhengtian, Jiasite, Aikang, and Chunli), while the Mobile-Bearing Group mainly uses the Oxford Phase III system and its licensed derivative products. The heterogeneity in liner geometry, fixation methods, and manufacturing tolerances within the Fixed-Bearing Group may introduce system related biases, and the relatively small sample size of each specific prosthesis hinders meaningful subgroup analysis. This should be considered as a potential source of variation.

In summary, in this elderly patient cohort, the Mobile-Bearing unicompartmental is associated with better mid-term pain control, functional recovery, and patient reported quality of life, although the clinical significance of these differences remains to be fully determined. Its biomechanical design promotes more physiological joint movement and force line recovery. However, these clinical benefits must be carefully weighed against significantly higher medical costs and specific risk characteristics of complications. The Fixed-Bearing unicompartmental provides an economically more advantageous and reliable alternative with a different spectrum of complications. Given the retrospective design and limited absolute effect size, these observed associations should not be interpreted as definitive causal evidence. Therefore, the final clinical decision should be individualized, integrating patient specific factors such as chronological age and biological age, activity expectations, bone mass, ligament integrity, and payment ability. The shared decision-making framework is crucial for developing the optimal treatment path and maximizing the overall value of medical services.

## Data Availability

The raw data supporting the conclusions of this article will be made available by the authors upon reasonable request, subject to the following restrictions: (1) Institutional Review Board approval requirements, (2) patient privacy and confidentiality protections under Chinese healthcare regulations, (3) data use agreements with the four participating joint centers, and (4) removal of personally identifiable information. Requests for data access should be directed to the corresponding author.

## References

[1] Migliorini F Maffulli N Cuozzo F Elsner K Hildebrand F Eschweiler J. Mobile bearing versus fixed bearing for unicompartmental arthroplasty in monocompartmental osteoarthritis of the knee: a meta-analysis. J Clin Med. (2022) 11(10):2837. 10.3390/jcm11102837 35628963 PMC9143434

[2] Hiranaka T. Advantages and limitations of mobile-bearing unicompartmental knee arthroplasty: an overview of the literature. Expert Rev Med Devices. (2024) 21(7):587–600. 10.1080/17434440.2024.2367002 38873929

[3] Cao Z Niu C Gong C Sun Y Xie J Song Y. Comparison of fixed-bearing and mobile-bearing unicompartmental knee arthroplasty: a systematic review and meta-analysis. J Arthroplasty. (2019) 34(12):3114–23.e3. 10.1016/j.arth.2019.07.005 31474324

[4] Bonano JC Barrett AA Amanatullah DF. Medial unicompartmental knee arthroplasty with a mobile-bearing implant. JBJS Essent Surg Tech. (2021) 11(2):e20.00002. 10.2106/JBJS.ST.20.00002 34277135 PMC8280033

[5] Kawaguchi K Inui H Taketomi S Yamagami R Nakazato K Shirakawa N. Intraoperative mobile-bearing movement in Oxford unicompartmental knee arthroplasty. Knee Surg Sports Traumatol Arthrosc. (2019) 27(7):2211–7. 10.1007/s00167-018-5064-6 30030580

[6] Fricka KB Wilson EJ Strait AV Ho H Hopper RH Jr Hamilton WG. Outcomes of fixed versus mobile-bearing medial unicompartmental knee arthroplasty. Bone Joint J. (2024) 106-B(9):916–23. 10.1302/0301-620X.106B9.BJJ-2024-0075.R1 39216863

[7] Sun X Liu P Lu F Wang W Guo W Zhang Q. Bearing dislocation of mobile bearing unicompartmental knee arthroplasty in east Asian countries: a systematic review with meta-analysis. J Orthop Surg Res. (2021) 16(1):28. 10.1186/s13018-020-02190-8 33413535 PMC7791981

[8] Smith E Lee D Masonis J Melvin JS. Lateral unicompartmental knee arthroplasty. JBJS Rev. (2020) 8(3):e0044. 10.2106/JBJS.RVW.19.00044 32149936

[9] Morales-Avalos R Perelli S Raygoza-Cortez K Padilla-Medina JR Peña-Martínez VM Guzmán-López S. Fixed-bearing unicompartmental knee arthroplasty provides a lower failure rate than mobile-bearing unicompartimental knee arthroplasty when used after a failed high tibial osteotomy: a systematic review and meta-analysis. Knee Surg Sports Traumatol Arthrosc. (2022) 30(9):3228–35. 10.1007/s00167-021-06707-4 34415370

[10] Song MH Kim KT Hwang YS Kim JW Eom TW Chae JH. Late mobile-bearing dislocation in unicompartmental knee arthroplasty. Orthopedics. (2019) 42(1):e124–7. 10.3928/01477447-20181010-09 30321443

[11] Nan S Cao Z Song Y Kong X Li H Chai W. Can mobile-bearing unicompartmental knee arthroplasty achieve natural gap-balancing? An observational study with a novel pressure sensor. J Orthop Surg Res. (2022) 17(1):407. 10.1186/s13018-022-03255-6 36064425 PMC9446724

[12] Suda Y Hiranaka T Kamenaga T Fujishiro T Okamoto K Matsumoto T. Mobile bearing orbit on the tibial component in Oxford unicompartmental knee arthroplasty. Knee. (2023) 42:136–42. 10.1016/j.knee.2023.03.003 37001330

[13] Crawford DA Rutledge-Jukes H Alexander JS Lombardi AV Jr Berend KR. 15-year follow-up of mobile bearing medial unicompartmental knee arthroplasty. J Arthroplasty. (2023) 38(7):1257–61. 10.1016/j.arth.2023.01.024 36708937

[14] Yang I Agustoni G Murray DW Mellon SJ. Mechanisms of mobile bearing dislocation in lateral unicompartmental knee replacement. Proc Inst Mech Eng H. (2023) 237(10):1167–76. 10.1177/09544119231195678 37776125 PMC10634216

[15] Stempin R Stempin K Kaczmarek W. Medium-term outcome of cementless, mobile-bearing, unicompartmental knee arthroplasty. Ann Transl Med. (2019) 7(3):41. 10.21037/atm.2018.12.50 30906745 PMC6389574

[16] D'Ambrosi R Buda M Nuara A Mariani I Scelsi M Valli F. Patellar height after unicompartmental knee arthroplasty: comparison between fixed and mobile bearing. Arch Orthop Trauma Surg. (2022) 142(11):3449–60. 10.1007/s00402-021-04183-6 34669039

[17] Jennings JM Kleeman-Forsthuber LT Bolognesi MP. Medial unicompartmental arthroplasty of the knee. J Am Acad Orthop Surg. (2019) 27(5):166–76. 10.5435/JAAOS-D-17-00690 30407979

[18] Walker T Freericks J Mick P Trefzer R Lunz A Koch KA. Long-term results of lateral unicompartmental knee arthroplasty with a mobile-bearing device. Bone Joint J. (2025) 107-B(3):322–8. 10.1302/0301-620X.107B3.BJJ-2024-0859.R1 40020717

[19] Bonano JC Barrett AA Aggarwal VK Chen F Schirmers J Finlay AK. Supine knee positioning does not interfere with mobile-bearing unicompartmental knee arthroplasty performance. J Knee Surg. (2023) 36(10):1020–5. 10.1055/s-0042-1748822 35688441

[20] Kono K Inui H Tomita T Yamazaki T Taketomi S Yamagami R. Weight-bearing status affects in vivo kinematics following mobile-bearing unicompartmental knee arthroplasty. Knee Surg Sports Traumatol Arthrosc. (2021) 29(3):718–24. 10.1007/s00167-020-05893-x 32055876

[21] Hao D Wang J. Fixed-bearing vs mobile-bearing prostheses for total knee arthroplasty after approximately 10 years of follow-up: a meta-analysis. J Orthop Surg Res. (2021) 16(1):437. 10.1186/s13018-021-02560-w 34229702 PMC8259014

[22] Hantouly AT Ahmed AF Alzobi O Toubasi A Salameh M Elmhiregh A. Mobile-bearing versus fixed-bearing total knee arthroplasty: a meta-analysis of randomized controlled trials. Eur J Orthop Surg Traumatol. (2022) 32(3):481–95. 10.1007/s00590-021-02999-x 34021791 PMC8924090

[23] Jiang Y Liu C Zhang Q Sun G Ding R Zhang N. Restoring coronal pre-arthritic alignment in mobile-bearing unicompartmental knee arthroplasty: mid- to long-term outcomes. BMC Musculoskelet Disord. (2025) 26(1):124. 10.1186/s12891-025-08363-y 39915792 PMC11800512

[24] Jiao X Li Z Du M An S Huang J Cao G. Valgus stress radiograph can predict alignment change of medial mobile-bearing unicompartmental knee arthroplasty. Orthop Surg. (2023) 15(7):1847–53. 10.1111/os.13823 37395116 PMC10350385

[25] Sun X Wang Q Ge J Wang W Guo W Zhang Q. Sensor assessment of gap balance in mobile-bearing unicompartmental knee arthroplasty. J Vis Exp. (2022) (185):e63711. 10.3791/63711 35913146

[26] Shen X Zhang X Liu Y Zhu C Huang W. Challenges in residual bearing removal: a rare case of mobile bearing fracture in unicompartmental knee arthroplasty with literature review. Orthop Surg. (2024) 16(8):2087–92. 10.1111/os.14138 38946660 PMC11293902

[27] Buzin SD Geller JA Yoon RS Macaulay W. Lateral unicompartmental knee arthroplasty: a review. World J Orthop. (2021) 12(4):197–206. 10.5312/wjo.v12.i4.197 33959483 PMC8082511

[28] Wu K Lv G Yin P Dong S Dai Z Li L. Effect of tibial component overhang on survivorship in medial mobile-bearing unicompartmental knee arthroplasty. Knee. (2022) 37:188–95. 10.1016/j.knee.2022.06.011 35820266

[29] Lim S Kim TH Park DY Sunwoo J Chung JY. Mobile versus fixed-bearing in medial unicompartmental knee arthroplasty: an average 10-year follow-up. J Clin Med. (2025) 14(20):7144. 10.3390/jcm14207144 41156014 PMC12565152

[30] Migliorini F Maffulli N Cuozzo F Pilone M Elsner K Eschweiler J. No difference between mobile and fixed bearing in primary total knee arthroplasty: a meta-analysis. Knee Surg Sports Traumatol Arthrosc. (2022) 30(9):3138–54. 10.1007/s00167-022-07065-5 35861866 PMC9418337

[31] Crawford DA Berend KR Thienpont E. Unicompartmental knee arthroplasty: US and global perspectives. Orthop Clin North Am. (2020) 51(2):147–59. 10.1016/j.ocl.2019.11.010 32138853

[32] Kennedy JA Mohammad HR Yang I Mellon SJ Dodd CAF Pandit HG. Oxford domed lateral unicompartmental knee arthroplasty. Bone Joint J. (2020) 102-B(8):1033–40. 10.1302/0301-620X.102B8.BJJ-2019-1330.R2 32731833

[33] Yildiz F Incesoy MA Borjon ED Senyurt M Sheth N Alvand A. Is there a difference in clinical outcomes between fixed bearing and mobile bearing unicompartmental knee arthroplasty? J Arthroplasty. (2025) 40(2S1):S67–8. 10.1016/j.arth.2024.10.037 39428005

[34] Hiranaka T Suda Y Kamenaga T Fujishiro T Koide M Saitoh A. Bearing separation from the lateral wall of the tibial component is a risk of anterior dislocation of the mobile bearing in Oxford unicompartmental knee arthroplasty. J Arthroplasty. (2022) 37(5):942–7. 10.1016/j.arth.2022.01.020 35074447

[35] Ma J Zhang L Wang C Xu K Ren Z Wang T. The mid-term outcomes of mobile bearing unicompartmental knee arthroplasty versus total knee arthroplasty in the same patient. Front Surg. (2023) 10:1033830. 10.3389/fsurg.2023.1033830 36761029 PMC9905616

[36] Hariri M Zahn N Mick P Jaber A Reiner T Renkawitz T. Fixed-bearing is superior to mobile-bearing in lateral unicompartmental knee replacement: a retrospective matched-pairs analysis. Knee Surg Sports Traumatol Arthrosc. (2023) 31(9):3947–55. 10.1007/s00167-023-07417-9 37093235 PMC10435651

[37] Hoshi K Watanabe G Kurose Y Tanaka R Fujii J Gamada K. Mobile-bearing insert used with total knee arthroplasty does not rotate on the tibial tray during a squatting activity: a cross-sectional study. J Orthop Surg Res. (2020) 15(1):114. 10.1186/s13018-020-1570-6 32197628 PMC7085202

[38] Kang SW Kim KT Hwang YS Park WR Shin JK Song MH. Is mobile-bearing medial unicompartmental knee arthroplasty appropriate for Asian patients with the risk of bearing dislocation? J Arthroplasty. (2020) 35(5):1222–7. 10.1016/j.arth.2019.12.036 31952946

[39] Bae JH Kim JG Lee SY Lim HC In Y, MUKA Study group. Epidemiology of bearing dislocations after mobile-bearing unicompartmental knee arthroplasty: multicenter analysis of 67 bearing dislocations. J Arthroplasty. (2020) 35(1):265–71. 10.1016/j.arth.2019.08.004 31471182

[40] Zheng N Dai H Zou D Wang Q Tsai TY. Safe bearing region for avoiding meniscal bearing impingement and overhang in mobile-bearing unicompartmental knee arthroplasty. J Orthop Res. (2024) 42(6):1200–9. 10.1002/jor.25767 38084771

[41] D'Ambrosi R Valli F Nuara A Mariani I Di Feo F Ursino N. No difference in mobile and fixed bearing partial knee arthroplasty in octogenarians: a clinical trial. Eur J Orthop Surg Traumatol. (2023) 33(7):3081–8. 10.1007/s00590-023-03537-7 37017739 PMC10074352

[42] Zhang W Wang J Li H Wang W George DM Huang T. Fixed- versus mobile-bearing unicompartmental knee arthroplasty: a meta-analysis. Sci Rep. (2020) 10(1):19075. 10.1038/s41598-020-76124-z 33154502 PMC7645610

